# Plasma GDF15 level is elevated in psychosis and inversely correlated with severity

**DOI:** 10.1038/s41598-017-07503-2

**Published:** 2017-08-11

**Authors:** Parvin Kumar, Vincent Millischer, J. Carlos Villaescusa, Ida A. K. Nilsson, Claes-Göran Östenson, Martin Schalling, Urban Ösby, Catharina Lavebratt

**Affiliations:** 1 Department of Molecular Medicine and Surgery, Karolinska Institutet, Stockholm, Sweden; 2 Center for Molecular Medicine, Karolinska University Hospital, Stockholm, Sweden; 3 Department of Adult Psychiatry, PRIMA Barn och Vuxenpsykiatri AB, Stockholm, Sweden; 4 Department of Neurobiology, Care Sciences and Society, Karolinska Institutet, Stockholm, Sweden

## Abstract

Accumulating evidence suggests that GDF15 is a biomarker for ageing and morbidity of many somatic disorders such as cancer and inflammatory disorders. Recently, elevated serum GDF15 level was proposed as a marker for mood disorder. However, psychosis severity was not investigated in relation to plasma GDF15 levels. In the present study we measured GDF15 levels in plasma of 120 psychosis patients compared to 120 age and gender matched healthy controls. Within the patient cohort GDF15 levels were evaluated for association with age, gender, lifestyle factors, C-reactive protein levels, psychosis severity and metabolic disorder. Psychosis patients had elevated GDF15 levels compared to controls (median_Psychosis_ = 744 ng/mL, median_controls_ = 516 ng/mL, p < 0.001). Within the psychosis cohort, GDF15 levels, when corrected for age, metabolic health and lifestyle factors, were negatively correlated with psychosis severity (β = −0.218, p = 0.012). While GDF15 levels were elevated in patients versus healthy controls, the negative correlation between psychosis severity and GDF15 suggests a loss of anti-inflammatory GDF15 mediated functionality in severe psychosis. Study replication in larger cohorts will be necessary to assess the potential of GDF15 as a prognostic biomarker in psychosis.

## Introduction

Psychosis is a pathological trait present within psychiatric disorders such as the schizophrenia spectrum of disorders (SSD)^1^, delusional disorder^2^ and bipolar disorder (BD)^3^, which incapacitates sufferers from ascertaining reality^4^. SSD and BD share etiological determinants^5^, and are often accompanied by life-long social impairment^6^, decreased level of functioning and metabolic comorbidities, such as increased plasma fasting glucose levels and waist circumference^7, 8^. Elevated inflammatory markers have been reported in the peripheral blood of psychosis patients. In a meta-analysis, drug-naive first-episode psychosis (FEP) patients were found to have higher levels of interleukin-1β (IL-1β), interleukin-6 (IL-6), tumor necrosis factor alpha (TNF-α)^9^ and cardio-metabolic disturbances^10–12^. C-reactive protein (CRP), secreted by the liver in response to IL-1β, IL-6, and TNF-α has also been found to be elevated in psychotic disorders such as schizophrenia (SZ) and BD^13, 14^. The CRP levels in those groups were reported to be further increased by female gender, higher body mass index (BMI) and elevated blood glucose levels^14–18^.

The link between peripheral inflammation and metabolic comorbidity in psychotic disorders is unclear. Peripheral inflammation has been implicated in the activation of inflammatory networks in the central nervous system (CNS)^19^. Correspondingly, increased levels of pro-inflammatory cytokines and kynurenine metabolites were found in cerebrospinal fluid (CSF) and serum of chronic SZ patients in independent investigations^20, 21^. The extent to which peripheral inflammation influences CNS immune activation and vice-versa in different conditions is yet to be explored. However, irrespective of its origin, there is a growing body of support for inflammation having a significant role in the pathology and overall prognosis of psychotic disorders^22, 23^. Thus to better understand psychosis it is crucial to accurately describe the alteration of inflammatory markers.

Growth Differentiation Factor-15 (GDF15) is a member of the transforming growth factor-β (TGF-β) superfamily of cytokines, upregulated in response to injury with defined roles in regulating inflammation and apoptosis^24^. It is suggested to have an overall anti-inflammatory effect and is elevated in inflammatory scenarios associated with cancer, cardiovascular disease, insulin resistance and obesity^25^, as a compensatory mechanism^26–28^. GDF15 levels have been reported to correlate with CRP levels in certain inflammatory disorders^29, 30^. In addition to its growing relevance as a marker of inflammation^26^ and all-cause mortality^31^, GDF15 has been described in the literature to have neurotrophic^32, 33^ and anti-apoptotic properties^34^. A proteomics investigation for biomarkers in mood disorders conducted by Frye *et al*.^35^, described GDF15 as a putative biomarker for mood disorders, particularly BD type I. Four additional proteins had increased levels in BD type I compared to controls, but GDF15 had the strongest effect size^35^. The increased GDF15 levels were proposed to be driven by the disorder rather than by psychotropic medication. However, the authors stated that an assessment of psychotic features, which were particularly frequent in the BD type I group, could have provided greater understanding of the elevated GDF15 levels in BD type I.

Therefore, we have investigated GDF15 in the context of psychotic disorders with a focus on relating the GDF15 levels to psychosis severity. Given the previously reported relationship between circulating GDF15 levels and metabolic disorder^26–28^, also detected in Frye *et al*.^35^, we also considered metabolic comorbidity in the study.

The aims of the present study were to determine if plasma GDF15 levels (i) were different in psychosis patients compared to healthy controls, (ii) associated with severity of psychotic illness, and (iii) associated with degree of metabolic comorbidity and CRP levels in the patients. We hypothesized that plasma GDF15 levels would be (i) increased in psychosis, (ii) elevated by metabolic comorbidity, (iii) associated with CRP levels in patients.

## Materials and Methods

### Psychosis patient group

All patients in regular clinical treatment from several specialized psychosis clinics, mainly in Stockholm County, were as part of a general health screening asked to participate in the Swedish Study of Metabolic Risks in Psychosis (SMRP), approximately 1000 patients with psychosis were recruited. Clinical diagnoses of psychiatric illness were made according to the diagnostic and Statistical Manual of Mental Disorders, 4th Ed. (DSM-IV)^36^. Patients were assessed using psychiatric questionnaires with data on diagnosis, severity of mental illness reported by the clinician according to the Clinical Global Impression-Severity Index (CGI)^37^, duration of illness, type of medications and duration of treatment. Medication and dosage were confirmed by reference to medical records. Somatic health, including smoking status and alcohol consumption, was assessed with a self-reported questionnaire in the clinic under the supervision of a nurse. Body mass index and waist circumference were measured. The presence of psychiatric disease in first degree relatives was reported. Anti-psychotic pharmacotherapy along with all other variables used in analyses are detailed in Table 1. Venous blood, after an overnight fast, was collected. Whole blood and serum were stored at −80 °C until analysis. Three hundred of the patients participated in a follow-up, with identical protocol as at baseline, one year later. For the present investigation, 120 consecutively recruited patients, between 2005 and 2009, out of the 300 that had given blood samples at the one-year follow-up, were included in the study and data collected at the one year follow up were used (Tables 1 and 2). All participants gave written informed consent. Ethical approval for the study was obtained from the Stockholm Regional Ethics Review Board. All methods were performed in accordance with relevant guidelines and regulations stipulated by Karolinska Institutet and Stockholm Regional Ethics Review Board.

**Table 1:** Demographic and clinical characteristics of the psychosis patients (n = 120).

Patient characteristics	Median (IQR)	Range
Age [years]	53.6 (48.3–63.0)	44.2–82.4
Body Mass Index (BMI) [kg/m^2^]	28.4 (25.3–31.4)	17.4–51.3
Length of hospital stay [months]	5.0 (2.0–16.5)	0.0–120.0
Alcohol consumption [g/week]	15.0 (0.0–76.0)	0–1176
Age at onset [year]	28.0 (23.0–39.5)	10–65
Duration of anti-psychotic treatment [years]	22.5 (14.0–29.0)	2–56
Clinicians Global Impression (CGI)- Severity	3 (3–4)	1–7
	**n**	**%**
Gender		
Male	62	51.7
Female	58	48.3
Psychosis characteristics		
Early psychosis onset (≤18 yr old)	109	90.8
Late onset (>18 yr old)	11	9.2
Smoking		
Yes (every day or sometimes)	36	30.0
No (never or quit)	62	51.7
Unknown	22	18.3
Presence of psychiatric disease in first-degree relative		
Yes	48	40.0
No	72	60.0
Main psychiatric diagnose		
Schizophrenia	45	37.5
Schizoaffective disorder	23	19.2
Delusional disorder	5	4.2
Psychosis unspecified (UNS)	4	3.3
Bipolar disorder	13	10.8
Other	9	7.5
Undiagnosed psychosis	21	17.5
Antipsychotic medication		
None	24	20.0
Multitherapy	9	7.5
Risperidone	19	15.8
Olanzapine	25	20.8
Zuclopenthixol	10	8.3
Perphenazine	13	10.8
Haloperidol	11	9.2
Clozapine	5	4.2
Aripiprazole	9	7.5
Quetiapine	6	5.0
Ziprasidone	3	2.5
Flupentixol	5	4.2

**Table 2:** Metabolic characteristics of the psychosis patients (n = 120).

Metabolic characteristics	Median (IQR)	Range	Reference
Men			
Waist circumference [cm]	102 (95–113)	69.0–143.0	<94.0
Triglycerides [mmol L^−1^]	1.4 (0.95–2.00)	0.47–4.50	<1.70
HDL-cholesterol [mmol L^−1^]	1.00 (0.90–1.20)	0.50–2.30	>1.03
LDL-cholesterol [mmol L^−1^]	3.30 (2.50–3.83)	1.50–5.30	<2.6
Fasting plasma glucose [mmol L^−1^]	5.30 (5.00–5.90)	4.60–17.50	<5.60
Log HOMA - IR	0.637 (0.489–0.916)	0.10–1.76	<2.00
Women			
Waist circumference [cm]	99.0 (88.5–110)	77.5–142.0	<80.0
Triglycerides [mmol L^−1^]	0.925 (1.20–1.95)	0.41–2.70	<1.70
HDL-cholesterol [mmol L^−1^]	1.40 (1.05–1.60)	0.60–2.40	>1.29
LDL-cholesterol [mmol L^−1^]	3.70 (3.00–4.60)	0.60–2.40	<2.6
Fasting plasma glucose [mmol L^−1^]	5.30 (5.00–5.80)	4.50–7.90	<5.60
Log HOMA - IR	0.594 (0.424–0.763)	0.22–1.18	<2.00

IQR: interquartile range.

HDL: high-density-lipoprotein.

LDL: low-density-lipoprotein.

Log HOMA-IR: log_10_ of homeostatic model of assessment for insulin resistance.

GDF15 levels in patient plasma were tested for association with age, gender, CGI, smoking status, alcohol use [g/week], as well as with variables describing the metabolic profile, levels of low-density-lipoprotein (LDL), high-density-lipoprotein (HDL), fasting glucose and insulin sensitivity marker homeostatic model of assessment for insulin resistance (HOMA-IR)^38^. CRP was used to indicate inflammation. Gender and smoking were dichotomous variables while the other variables were continuous. Non-smokers were those who did not smoke at all and all others were considered as smokers.

### Healthy controls

Control subjects were selected from the population based Stockholm Diabetes Prevention Program (SDPP) which comprised ~5700 participants recruited during 1992–1998 who were followed-up eight to ten years later (2002–2006)^39^. A total of 120 unrelated Swedish SDPP subjects were selected by matching with the patients for age, gender and BMI. All subjects had normal glucose tolerance at baseline and follow-up and a family history of diabetes corresponding to the population average in Sweden. Of the 120 control subjects 62 (51.7%) were males and 58 (48.3%) were females. The median and interquartile range (IQR) of age of the controls was 53.5 (48.0–61.3) years. The BMI median and IQR of the controls was 28.4 (25.3–31.2) kg/m^2^. Of the 120 control subjects 29 (24.1%) had family history of diabetes at baseline and 30 (25.0%) had family history of diabetes at follow-up. The samples from healthy controls were collected at the same time point as patient samples, at the one year follow up of the study and treated in an identical manner.

### Measurement of routine metabolic and inflammation markers in the SZ patients

Serum lipid profiles (HDL and LDL), plasma glucose and insulin levels were determined according to standard GLP protocols. High-sensitive C-reactive protein (hsCRP) levels were determined in serum using near infrared particle immunoassay (NIPIA). All analyses were done at the Karolinska University Hospital (KUH) Laboratory in accordance with KUH stipulated good clinical practices and regulations. Reference range forhsCRP was as follows, Low risk: less than 1.0 mg/L. Average risk: 1.0 to 3.0 mg/L. High risk: above 3.0 mg/L.

### Measurement of GDF15

The plasma samples were assayed for GDF15 protein levels with the Quantikine^®^ ELISA Human GDF15 immunoassay (R&D Systems) according to the manufacturer’s instructions. A subset of the samples were assayed with technical replicates. The optical density of sample wells was determined using the Microplate Reader by Thermo Labsystemsm Inc, and the software Multi-skan Ascent^TM^ for processing the data. The GDF15 concentration of each sample was calculated using the measured absorbance against a standard curve. All samples had been freeze-thawed only once prior to analysis and all samples were run within the same week.

### Statistical analyses

Analysis of covariance of GDF15 levels in plasma was carried out to ascertain the effect of psychiatric diagnosis on GDF15 levels when corrected for age and lifestyle factors known to affect GDF15 levels. Two-group comparison (psychosis patients versus controls) of GDF15 levels was performed using non-parametric Mann-Whitney U test. CGI and GDF15 levels were normalized using natural logarithmic transformation (ln). Correlation analyses to test for association between GDF15 levels and the independent variables age and hsCRP were performed using Spearman’s rank correlation test.

To study putative predictors of plasma GDF15 levels, Ln GDF15 was assigned as a dependent variable and linear regression was performed using a stepwise method. The stepwise model is a semi-automated process of building a regression model based on the probability value of the F-statistic (p value) of the predictor regression coefficients^40^. We applied a stepwise iterative method of regression modelling starting with no variables in the model, testing the addition of each variable with an entry requirement of p < 0.05 and a loss of significance at p > 0.10. The distributions of regression residuals were checked to ensure normality and avoid multicollinearity.

Three linear regression models were constructed to ascertain the effects of 1) demography, psychosis and lifestyle 2) metabolic factors and 3) drug treatment on plasma GDF15 levels of the patients. The equation looking at demographic, psychosis and life style factors was as follows: Ln GDF15 = b_0_ + b_1_ (age) + b_2_ (gender) + b_3_ (lnCGI) + b_4_ (smoking) + b_5_ (alcohol). The second equation looking at metabolic factors was constructed as follows: Ln GDF15 = b_0_ + b_1_ (age) + b_2_ (gender) + b_3_ (waist) + b_4_ (LDL) + b_5_ (HDL) + b_6_ (glucose) + b_7_ (Log HOMA-IR). The third equation which analyzed the effect of medication on GDF15 levels was constructed as follows: Ln GDF15 = b0 + b1(age) + b2 (gender) + b_n_(drug), where the presence or absence of n = 11 different anti-psychotic medications which patients of the cohort were treated with were tested for effects on GDF15. The number of patients on each drug is detailed in Table 1.

To compare the effects of metabolic factors to the effects of lifestyle, demographic and psychosis-severity factors we imported the significant variables from the three models to a fourth regression model and exposed it to the forward stepwise method. The equation of the model was as follows: Ln GDF15 = b0 + b1 (age) + b2 (gender) + b3 (lnCGI) + b4 (smoke) + b5 (HDL). Analyses were carried out using IBM SPSS Statistics version 23, (IBM Corporation, USA).

## Results

Characteristics of the psychosis patient group are shown in Tables 1 and 2. Psychiatric diagnose categories in the psychosis patient group were schizophrenia, schizoaffective disorder, delusional disorder, psychosis unspecified, bipolar disorder and other. We assessed the effect of diagnosis on GDF15 levels using an analysis of covariance correcting for covariates proposed to influence GDF15 levels, that is age and smoking. There was no difference in mean GDF15 levels between the psychosis diagnosis groups (p = 0.260), therefore the psychosis patient group was treated as one group in the following analyses.

**Figure 1:**
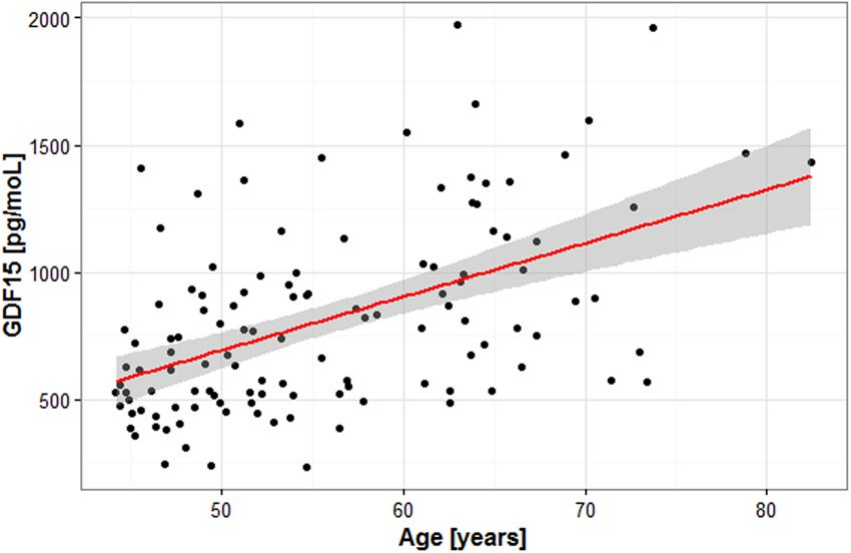
Plasma GDF15 levels are correlated with age in psychosis patients. Spearman’s ρ = 0.496, β_Standardized_ = 0.494, p < 0.001.

Psychosis patients had significantly higher plasma GDF15 levels compared to healthy controls: median_patients_ = 744 ng/mL, median_controls_ = 516 ng/mL, p < 0.001 (Table 3). Forty psychosis patients (33%) had hsCRP levels above high risk reference, greater than 3.0 mg /L, median_Ps_ = 2.05 mg/L [IQR: 0.923–4.00 mg/L]. No correlation was observed between GDF15 and hsCRP levels (Spearman’s ρ = 0.148, p = 0.11, n = 120, Standardized β = 0.123). In the psychosis group and healthy controls, the GDF15 plasma levels were significantly correlated with age at sampling (Fig. 1). Psychosis patients: Spearman’s ρ = 0.496, β_Standardized = _0.494, p < 0.001. Healthy controls: Spearman’s ρ = 0.342, β_Standardized_ = 0.338, p < 0.001.

**Table 3:** GDF15 and high-sensitive C-reactive protein (hsCRP) levels in psychosis patients and healthy controls

	Patients (Inter-quartile range) Range	Controls (Inter-quartile range) Range
GDF15 [pg/mL]**	744 (528–1008) 232–1972	516 (444–608) 267–1164
hsCRP [mg/moL]	2.05 (0.923–4.00) 0.00–18.9	NA

**p < 0.001

**Figure 2:**
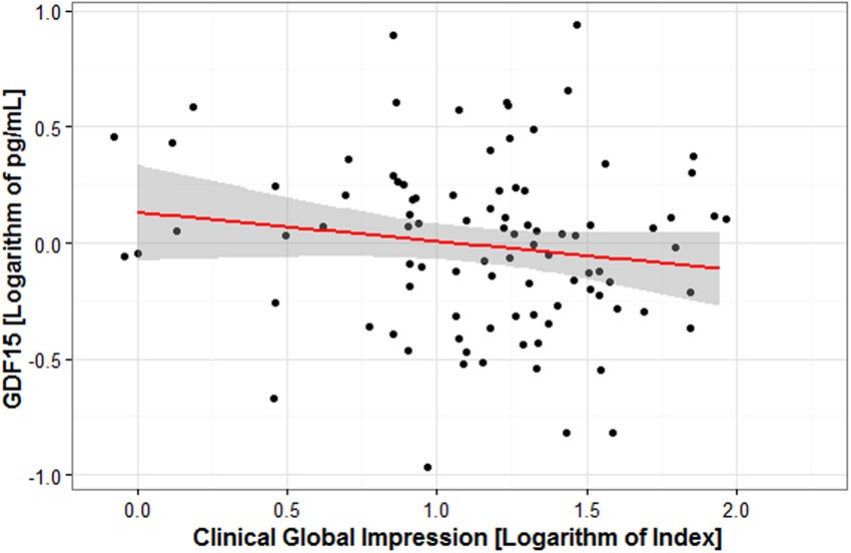
Plasma GDF15 levels are correlated with psychosis severity measured as Clinical Global Impression index (CGI) in psychosis patients. Higher CGI scores, representing more severe psychosis, were associated with lower GDF15 levels (β_standardized_ = −0.221, t = −2.58, p = 0.012.

Association between GDF15 levels and severity of psychotic illness was assessed within the psychosis group through the severity score CGI. A multiple linear regression model constructed in a stepwise way, including the predictors: age, CGI, gender, alcohol use and smoking, returned all predictors but alcohol use as statistically significant. The model explained 37.8% of the variance in GDF15 levels (adjusted R^2^ = 0.378, F = 14.8, n = 91). Higher CGI scores (more severe psychosis) were associated with lower GDF15 levels (β_standardized_ = −0.221, t = −2.58, p = 0.012) (Fig. 2). Increasing age was associated with higher GDF15 levels (β_standardized_ = 0.491, t = 5.49, p < 0.001). Smoking was associated with higher GDF15 levels (β_standardized_ = 0.313, t = 3.75, p < 0.001). The male gender was associated with increased GDF15 levels (β_standardized_ = −0.176, t = −2.00, p = 0.049).

GDF15 levels were tested for association with metabolic profile through waist circumference, LDL, HDL, fasting blood glucose and HOMA-IR levels. The multiple linear regression model constructed in a stepwise method to identify putative metabolic predictors of GDF15 levels, was adjusted for age and gender. Age and HDL levels were returned as significant variables in a model that explained 30.9% of the variance in GDF15 levels (adjusted R^2^ = 0.309, F = 22.5, n = 96). Higher age was associated with higher GDF15 values (β_standardized_ = 0.590, t = 6.661, p < 0.001). Higher HDL levels were associated with lower GDF15 levels (β_standardized_ = −0.234, t = −2.643, p = 0.01). The variables gender, waist circumference, LDL, glucose and HOMA-IR levels were not associated with GDF15 levels.

The effect of anti-psychotic medication on circulating GDF15 levels was assessed through multiple linear regression constructed in a stepwise method. The model was constructed to include age, gender, smoking status and drug treatment. Significant variables in this model that explained 33.6% of the variance in GDF15 levels (adjusted R^2^ = 0.336, F = 25.5, n = 97) included age (β_standardized_ = 0.490, t = 5.92, p < 0.001), and smoking status (β_standardized_ = 0.338, t = 4.09, p < 0.001) but there was no significant contribution from variables indicating drug treatment.

A final model was created to compare metabolic effects with lifestyle, demographic and psychosis severity factors in their contribution to GDF15 level variance. The model included the factors: age, smoking, gender, CGI and LDL and we found that the variables age, smoking, gender and lnCGI could explain 35.9% of the variance in GDF15 levels (adjusted R^2^ = 0.359. ANOVA: F = 13.8, n = 95, p < 0.001). Higher age was associated with higher GDF15 levels (β_standardized_ = 0.514, t = 5.81, p < 0.001). Smoking was associated with higher GDF15 values (β_standardized_ = 0.269, t = 3.22, p < 0.001). Higher CGI, an indication of more severe psychosis, was associated with lower GDF15 levels (β_standardized_ = −0.188, t = −2.55, p = 0.012). HDL levels did not improve subsequent model building and thus were omitted by the regression method from the final model. The results from all regression analyses are summarized in Table 4

**Table 4:** Summary of results from linear regression analyses on natural logarithm (ln) of plasma GDF15 levels.

Independent Variables	B	SE	β _standardized_	t	p
Model 1 (adjusted R^2^ = 0.378, ANOVA: F = 14.8, n = 91)					
**(Constant)**	4.13	0.294		14.1	0.001
**Age**	0.250	0.005	0.491	5.49	0.001
**Smoking**	0.300	0.080	0.313	3.75	0.001
**Gender**	−0.163	0.081	−0.176	−2.00	0.049
**Ln CGI**	−0.197	0.076	−0.221	−2.58	0.012
Model 2 (adjusted R^2^ = 0.309, ANOVA: F = 22.5, n = 96)					
**(Constant)**	3.83	0.256		15.197	0.001
**Age**	0.030	0.005	0.590	6.661	0.001
**HDL**	−0.274	0.097	−0.234	−2.643	0.01
Model 3 (adjusted R^2^ = 0. 336, ANOVA: F = 25.5, n = 97)					
**(Constant)**					
**Age**	0.026	0.044	0.490	5.92	<0.001
**Smoke**	0.327	0.082	0.338	4.09	0.001
Model 4 (adjusted R^2^ = 0.359, ANOVA: F = 13.8, n = 95)					
**(Constant)**	4.11	0.293		14.0	0.001
**Age**	0.026	0.005	0.514	5.81	0.001
**Smoking**	0.252	0.078	0.269	3.22	0.002
**Gender**	−0.171	0.079	−0.188	−2.15	0.034
**Ln CGI**	−0.194	0.076	−0.218	−2.55	0.012

CGI: Clinical Global Impression-Severity.

HDL: high-density-lipoprotein.

.

## Discussion

GDF15 has been implicated in the pathology of various disorders, with both adverse and beneficial effects depending on the cells’ state and microenvironment^41–44^. Its elevated level in inflammatory states is suggested to be due to a compensatory anti-inflammatory effect^26, 28^. In the present study, we show for the first time that GDF15 is elevated in plasma of psychosis patients when compared to healthy age and gender matched controls. In addition, we show in our psychosis patient cohort, that GDF15 levels are lower in patients with a more severe psychosis. A potential description of its role as a protective agent was previously proposed in the context of cardiovascular disease, obesity and inflammatory response *in vivo* ^28, 45–47^. GDF15 has been investigated in the context of neurodegeneration, dementia, BD type I and II and unipolar depression, and found to be elevated in plasma of these patient groups compared to healthy controls^35, 48–50^. Some patients with BD, especially BD type I, have episodes of psychosis, but GDF15 levels have not previously been studied specifically in relation to psychosis. Psychosis is associated with accelerated cellular ageing, and the life expectancy in psychosis patients is decreased by 15–17 years^51^. Our finding, that GDF15 is increased in psychosis patients, is in line with the notion that GDF15 concomitantly increases with morbidity and all-cause mortality^31, 35, 52^.

In order to be able to propose GDF15 as a potential prognosis biomarker in psychosis, further studies delineating the role of GDF15 in CNS pathways, especially where they intersect with the pathogenesis of psychosis, are required. In mammals it is known that GDF15 is produced by the placenta, liver, kidney, heart and CNS. Additionally it is secreted by the choroid plexus (ChP) into the CSF by the ChP where it serves as a neurotrophic, anti-apoptotic and anti-inflammatory molecule^34, 43^.

The serotonergic and dopaminergic systems have been implicated in psychosis^53, 54^, therefore it is of specific interest that Gdf15 promotes survival and differentiation of embryonic rat dopaminergic neurons and serotonergic raphe neurons. Strelau *et al*., have shown that Gdf15 is neurotrophic and neuroprotective to rat neurons *in vitro* and *in vivo*. They described that Gdf15 rescued intoxicated dopaminergic neurons of the 6-hydroxydopamine (6-OHD) model of Parkinsonism^32^. In a mouse study using *Gdf15* knockout mice they showed that Gdf15 is neurotrophic to sensory and motor neurons^33^. More recently Macahado *et al*., have described the importance of Gdf15 in the survival of dopaminergic neurons through a similar model of Parkinsonism as in Strelau *et al*., showing that *Gdf15* knockout mice are more severely affected than the wild-types with a greater loss of dopaminergic neurons in response to 6-OHD intoxication^55^.

A growing body of evidence suggests that inflammation plays a role in psychosis etiology and disease progression. An increased expression of astrocyte markers was observed in the grey matter of SZ patients suggestive of neuroinflammation^56^. Neuronal deficit caused by increased apoptosis, precipitated by microglial activation and pro-inflammatory cytokine release, has been linked to the neurodegenerative effects observed in SZ patients such as white matter disorders^57^. The association between SZ related neurodegeneration, and cognitive decline has been established by a long-term follow up investigation^58^. Interestingly, GDF15 levels have been found to be elevated in cognitive decline and age-related dementia^48, 49, 59^. GDF15 levels have been reported to be elevated in multiple inflammatory scenarios and found to be concomitantly increased with astrocytic gliosis^26, 44^. Lastly, GDF15 regulates leukocyte recruitment to areas of inflammation through regulating leukocyte integrin activation^46^. Taken together this could suggest that a role of GDF15 in SZ pathogenesis is related to preventing inflammation and neuro-degeneration.

The observation of a negative relationship between GDF15 and psychosis severity (CGI) within our psychosis cohort, is seemingly at odds with our finding that GDF15 was elevated in the patients compared to healthy controls, and the consensus of literature that higher GDF15 level is associated with increased disease morbidity. A plausible explanation for this could be that GDF15 is anti-inflammatory and thus higher levels of GDF15 could contribute to milder psychosis related co-morbidity and improved outcomes. GDF15 has been reported to have a beneficial effect in *in vitro* and *in vivo*. Gdf15 is released upon myocardial infarction, inhibiting polymorphonuclear leukocyte recruitment and inflammatory response which reduced the rate of pathology after myocardial infarction in rodents^45–47^, and increased production of Gdf15 led to smaller atherosclerotic lesions in the *ApoE* ^−/−^ mouse model of atherosclerosis^28^. Transgenic mice expressing human GDF15 were found to have reduced white adipose tissue and inflammatory response, a potential indication of protection against the inflammatory metabolic syndrome^47^. Apart from the dopaminergic and serotonergic neurons, Gdf15 has been shown to have a neurotrophic effect on the motor and sensory neurons^32–34^. Our finding that GDF15 levels were lower in the more severely afflicted psychosis patients could reflect the loss of anti-inflammatory and neuroprotective capabilities in these patients.

While in this study we have analyzed patient plasma for GDF15 and correlated this with psychosis severity, analyzing the CSF could prove to be a more attractive biological sample for the purpose of understanding the role of GDF15 in psychosis. In a study looking at glioblastoma, Shnaper S *et al.* have shown that elevated levels of GDF15 in the CSF is associated with worse patient outcomes indicating that our findings in patient plasma needs to be contrasted with CSF GDF15 levels^42^. In a separate study Kim DH *et al*., showed that Gdf15 delivered through the CSF promotes hippocampal neurogenesis and synaptic activity in an *in vivo* Alzheimer’s disease model. While we found that plasma GDF15 levels were lower in the patients who had higher psychosis severity scores, the CSF GDF15 levels could in fact be elevated in the worst outcomes by ChP mediated filtration of GDF15 from systemic circulation to the CNS marking a shift in its relative prevalence aimed at conferring a protective and compensatory function to the CNS.

CRP is an indicator of inflammation which can be present at chronic low level in psychosis patients especially in those with metabolic comorbidity, which is common particularly in those who undergo certain anti-psychotic drug treatments^60^. In the present study we did not detect a significant correlation between GDF15 levels and CRP levels within the psychosis patients. Multiple meta-analyses have shown that CRP levels are moderately elevated in SZ and BD, and, the elevated levels are increased by age, BMI and hyperglycemia^16, 18^, as GDF15 levels are^27, 61^. However, sex dependence seems different between CRP and GDF15 plasma levels, with lower CRP levels and higher GDF15 levels in males^15, 62^. This, together with that plasma CRP is secreted mainly by hepatocytes, that is, cells different from those secreting plasma GDF15, possibly contributes to the poor correlation between GDF15 and hsCRP^32, 43^. Also acute inflammation may blunt an association between GDF15 and CRP levels. Accordingly, the limited studies that looked at both GDF15 and CRP levels in the disorders chronic obstructive pulmonary disease^29^, patients undergoing hemodialysis^30^, subclinical atherosclerosis^63^, anaemia^64^ and rheumatoid arthritis^65^ have arrived at discrepant results, where the GDF15-CRP correlations vary from strongly statistically significant in the former two studies to non-significant in the latter studies^65^. To our knowledge our study is the first to compare these inflammation related markers in psychosis patient plasma. Age, gender, smoking and metabolic disorder have been associated with increased GDF15 levels in plasma or tissue specific GDF15 secretion^27, 31, 61, 62, 66^. Accordingly, we found positive associations for GDF15 levels to age, male gender and smoking. However, in our cohort the metabolic factors were relatively less important than the psychosis severity index in explaining GDF15 levels. In agreement with findings in mood disorder patients^35^, we detected no effect of type of antipsychotic medication on the GDF15 levels. However, the sample size was small for certain drugs. Thus, our findings propose that plasma GDF15 levels capture a process partly different from inflammation indicated by CRP levels or metabolic comorbidity.

Although the patient cohort in this study is heterogeneous as their DSM-IV-based diagnoses suggest, all patients were recruited from psychosis-specialized outpatient clinics in Sweden. The patients had all been diagnosed with a severe psychosis and impaired functionality. Our choice to include all these psychosis patients followed the Research Domain Criteria (RDoC) paradigm^67^ which traverses clinical diagnoses by looking at domains of human behavior and/or functioning. We found no statistically significant differences in GDF15 levels between the DSM-IV-based diagnoses, however this may have been due to an inadequate sample size. In place of a symptom severity scale such as the positive and negative symptom scale^68^ (PANSS), which was unavailable to us in this study, we used the clinician-rated CGI-S, an indication of the patients’ mental health, psychosis and wellness. The lack of a symptom severity scale like PANSS is a main limitation of this study.

To conclude, we report that plasma GDF15 levels are elevated in chronic psychosis patients compared to healthy controls, and that the GDF15 levels are negatively correlated with psychosis severity. An assessment of plasma GDF15 levels in relation to psychosis has not previously been reported, thus our findings warrant replication efforts as well as an investigation of GDF15 levels as a prognostic marker for psychosis.

## Electronic supplementary material

Supplementary Information
