# Supplementary material for: Plasma GDF15 level is elevated in psychosis and inversely correlated with severity
Source: Sci Rep. 2017 Aug 11;7:7906. doi: 10.1038/s41598-017-07503-2 (PMC5554200; doi:10.1038/s41598-017-07503-2)

Supplementary file

Plasma GDF15 level is elevated in psychosis and inversely correlated with severity.

Parvin Kumar[1,2], Vincent Millischer[1,2], J. Carlos Villaescusa[1,2], Ida AK Nilsson[1,2], Claes-Göran Östenson[1], Martin Schalling[1,2], Urban Ösby[2,3,4], Catharina Lavebratt[1,2]

[1]Department of Molecular Medicine and Surgery, Karolinska Institutet, Stockholm, Sweden

[2]Center for Molecular Medicine, Karolinska University Hospital, Stockholm, Sweden

[3]Department of Adult Psychiatry, PRIMA Barn och Vuxenpsykiatri AB, Stockholm, Sweden

[4]Department of Neurobiology, Care Sciences and Society, Karolinska Institutet, Stockholm, Sweden.

Figure Legend

GDF15 values of peripheral plasma plotted against psychosis patients grouped according to psychosis severity as measured by Clinical Global Impression index (CGI). 'Healthy' refers to control volunteers and 'NA' refers to psychosis patients without information on CGI.

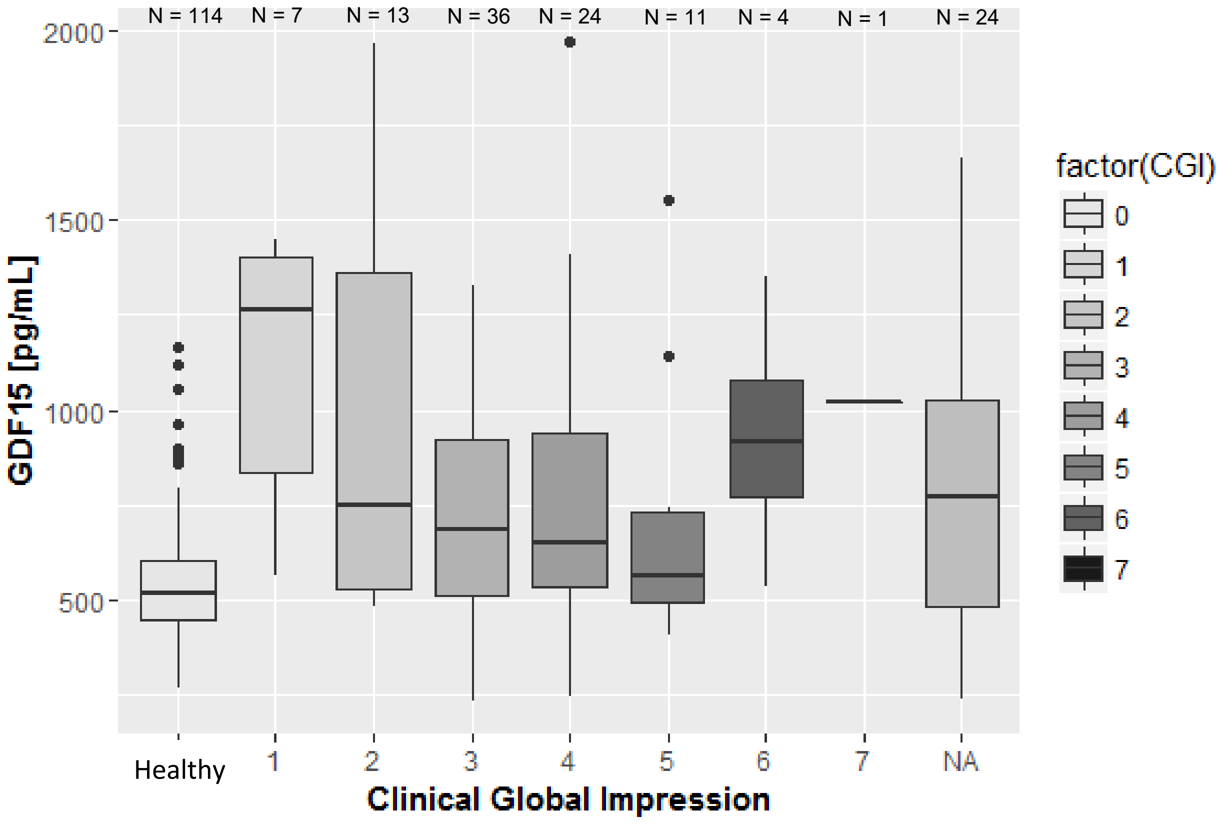

Supplement: Supplementary file 1 — Supplementary Information [file 41598_2017_7503_MOESM1_ESM.pdf]
